# Cellular Senescence in Human Aldosterone-Producing Adrenocortical Cells and Related Disorders

**DOI:** 10.3390/biomedicines9050567

**Published:** 2021-05-18

**Authors:** Jacopo Pieroni, Yuto Yamazaki, Xin Gao, Yuta Tezuka, Hiroko Ogata, Kei Omata, Yoshikiyo Ono, Ryo Morimoto, Yasuhiro Nakamura, Fumitoshi Satoh, Hironobu Sasano

**Affiliations:** 1 Department of Pathology, Tohoku University Graduate School of Medicine, Sendai 980-8575, Japan; jacopopieroni@gmail.com (J.P.); y.yamazaki@patholo2.med.tohoku.ac.jp (Y.Y.); gaoxin0222@dc.tohoku.ac.jp (X.G.); hiro37v5@gmail.com (H.O.); 2 Division of Clinical Hypertension, Endocrinology and Metabolism, Tohoku University Graduate School of Medicine, Sendai 980-8575, Japan; y.tezuka@med.tohoku.ac.jp (Y.T.); kei.omata.d4@tohoku.ac.jp (K.O.); y-ono@med.tohoku.ac.jp (Y.O.); fsatoh@med.tohoku.ac.jp (F.S.); 3 Division of Nephrology, Endocrinology, and Vascular Medicine, Tohoku University Hospital, Sendai 980-8575, Japan; rmorimoto@med.tohoku.ac.jp; 4 Division of Pathology, Faculty of Medicine, Tohoku Medical and Pharmaceutical University, Sendai 981-8558, Japan; yasu-naka@patholo2.med.tohoku.ac.jp

**Keywords:** immunohistochemistry, adrenal cortex, aldosterone, cellular senescence

## Abstract

In situ cortisol excess was previously reported to promote cellular senescence, a cell response to stress, in cortisol-producing adenomas (CPA). The aim of this study was to explore senescence pathways in aldosterone-producing cells and related disorders, and the influence of aldosterone overproduction on in situ senescence. We analyzed 30 surgical cases of aldosterone-producing adenoma (APA), 10 idiopathic hyperaldosteronism (IHA) and 19 normal adrenals (NA). CYP11B2 and senescence markers p16 and p21 were immunolocalized in all those cases above and results were correlated with histological/endocrinological findings. In the three cohorts examined, the zona glomerulosa (ZG) was significantly more senescent than other corticosteroid-producing cells. In addition, the ZG of adjacent non-pathological adrenal glands of APA and IHA had significantly higher p16 expression than adjacent non-pathological zona fasciculata (ZF), reticularis (ZR) and ZG of NA. In addition, laboratory findings of primary aldosteronism (PA) were significantly correlated with p21 status in *KCNJ5*-mutated tumors. Results of our present study firstly demonstrated that non-aldosterone-producing cells in the ZG were the most senescent compared to other cortical zones and aldosterone-producing cells in PA. Therefore, aldosterone production, whether physiological or pathological, could be maintained by suppression of cell senescence in human adrenal cortex.

## 1. Introduction

Cellular senescence is a complex, anti-proliferative cellular process which generally leads the cells to a state of irreversible growth arrest in response to different stressors, in order to remove the damaged cells from the homeostatic tissue environment [1]. Cellular senescence was also termed ‘replicative senescence’, which was perceived as the end of the replication program of diploid cells [2]. However, recent studies demonstrated that cellular mechanisms of senescence were actually more complex than previously considered and involved numerous different intracellular signal transduction pathways [3]. In particular, most senescence-inducing stressors have been reported to activate either the p16^Ink4a^ or p53/p21 intracellular signaling, which have emerged as possible biomarkers of cellular senescence [4,5]. p16^Ink4a^ was reported to be mostly involved in the senescence-induction process and p21 in the maintenance of cell cycle arrest [6].

The adrenal cortex is divided into three concentric zones: the outer ZG, the middle ZF, and the inner ZR, respectively each implicated in mineralocorticoids, glucocorticoids and androgens biosynthesis [7,8]. Aldosterone, the main mineralocorticoid, is biosynthesized in ZG cells in response to Angiotensin II or elevated plasmatic potassium concentration [9] and regulates intravascular volume and blood pressure, via sodium balance and fluid volume control [10]. An autonomous aldosterone overproduction results in PA, which could be either clinically unilateral, mainly in the form of APA, or bilateral as IHA [11,12]. Aldosterone biosynthesis has been reported to decrease with aging and is largely dependent on mitochondrial activities because of the localization of rate-limiting aldosterone biosynthetic enzyme, CYP11B2 (aldosterone synthase) [13,14]. The impact of aging on aldosterone production has been reported in the literature [15,16] but little is known about the cellular senescence of aldosterone-producing cells as well as the impact of aldosterone itself on the senescence process as well as its association with DNA damages. On the other hand, in situ cortisol excess was recently demonstrated to upregulate senescence markers in CPAs [17,18] possibly due to glucocorticoids-inducing stress response in the cells. Therefore, in this study, we attempted to explore the status of cellular senescence and γH2AX as a biomarker of DNA damage in physiological and pathological aldosterone-producing cells in order to analyze its clinicopathological significance in normal and PA adrenal glands.

## 2. Materials and Methods

### 2.1. Adrenals

A total of 30 APA and 10 IHA which had been surgically resected from 2012 and 2019 at Tohoku University Hospital, Sendai, Japan, were randomly selected from surgical pathology files of the Department of Pathology, Tohoku University School of Medicine, Sendai, Japan. In addition, 19 cases of NA, resected at the time of radical nephrectomy for renal cell carcinoma or other non-endocrine related diseases, were also retrieved from the files above and used as normal control. All PA cases were previously diagnosed according to the Japanese Endocrine Society Guidelines for PA [19]. The clinicopathological features of these 30 APA, 10 IHA and 19 NA cases are summarized in Supplemental Tables S1–S3. APAs were histologically diagnosed as adrenocortical adenomas according to Weiss’s criteria [20] and tumor size was calculated from the resected tissue specimens of adrenalectomy. This study protocol was approved by the IRB (Institutional Review Board) of Tohoku University School of Medicine (2020-1-316, 2020-1-477).

### 2.2. Immunohistochemistry (IHC) and Semi-Quantitative Analysis

All the specimens had been fixed in 10% neutral buffered formalin and embedded in paraffins. Serial tissue sections were made from 10% buffered formalin-fixed paraffin-embedded (FFPE) blocks cut at 3 μm thickness. Hematoxylin and eosin (H&E) staining and immunohistochemistry (IHC) were performed in these tissue sections. Methods of IHC employed in this study were summarized in Supplemental Table S4. All the stained slides were subsequently captured and digitally scanned using a virtual microscopy Image Scope AT2 (Leica, Wetzler, Germany). CYP11B2 IHC was used as immunohistochemical marker of aldosterone production [21]. p16, p21 and γH2AX was immunolocalized in the nuclei and analyzed using the digital image analysis software “HALO^TM^ CytoNuclear ver.1.5” using H-score or a labeling index (Indica Laboratories, Corrales, NM, USA) according to a previous report [22]. p16 and p21 were assessed in each adrenal cortical layer (zona glomerulosa, fasciculata and reticularis) and aldosterone-producing lesions. Among APA cases, ZG, ZF and ZR in the non-neoplastic or attached adrenals were termed ‘Adjacent’ (Adj.) and tumor cells were histologically classified into clear and compact tumor cells. Annotated target areas were determined via eyeball analysis and circumscribed after a careful evaluation of the whole histological section, in order to further confirm that those areas were representative of the entire sections (to avoid the influence of intratumoral heterogeneity as much as possible). Immuno-positive cells were tentatively classified into the following four categories according to their relative immune-intensity: negative as “0”, weak (yellow) as “+1”, moderate (orange) as “+2”, and strong (red) as “+3” and the H-score was subsequently obtained as the number of the individual gradients of the positive cells X Score 1+, 2+, 3+)/Total cells X100 [23,24].

### 2.3. Microdissection and DNA Extraction from FFPE Blocks

Another subset of serial tissue sections of APA cases was prepared as follows: H&E (3 μm), CYP11B2 (3 μm), 8 unstained sections at 10 μm thickness for DNA extraction, CYP11B2 (3 μm). Then, CYP11B2-positive areas were individually microdissected from the unstained sections in corresponding areas according to the results of CYP11B2 immunolocalization, and genomic DNA for PCR analysis was extracted using the AllPrep^®^ DNA FFPE Kit (QIAGEN, Tokyo, Japan) as previously described [25]. All 30 APA cases were further examined for the presence or absence of *KCNJ5* somatic mutation using sanger sequencing, as previously reported [26,27]. *KCNJ5* somatic mutation was detected in 18 cases and 12 were considered WT.

### 2.4. Statistical Analysis

The comparison of immunoreactivity and clinicopathological factors among these cases above were analyzed using Mann Whitney’s U test and Kruskal‒Wallis’s test. In multiple comparison analyses, a pairwise post-hoc analysis was performed using Dunn’s test. The Spearman’s test was used to evaluate the statistical correlations. *p*-values < 0.05 were considered significant. The tests were performed with the software “JMP Pro ver. 15.0.0” and “R^2^-statistics”.

## 3. Results

### 3.1. Immunolocalization of Cell Senescence Markers in NA, APA and IHA

**Figure 1:**
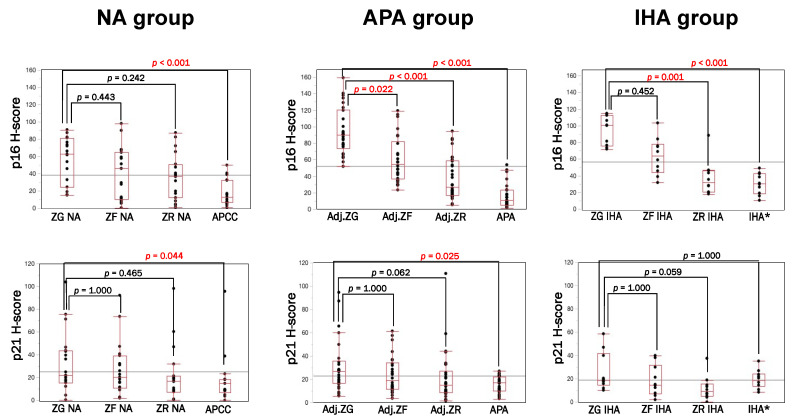
p16, p21 in NA, APA and IHA cohort. p16 IHC revealed that ZG in the APA and IHA group was significantly more senescent than ZF, ZR and aldosterone-producing lesions. In the NA group, ZG was higher than ZF. Results of p21 were equivalent to those of p16 but there were some differences in the three cohorts. (*) is used to identify the CYP11B2-positive lesions responsible for aldosterone overproduction in the IHA group.

As summarized in Figure 1, in NA, p16 and p21 in ZG were significantly higher than aldosterone-producing cell clusters (APCC) (*p* < 0.001; *p* = 0.044), but there were no significant differences between ZG and ZF or ZR of NA. In APA, p16 was predominantly immunolocalized in Adj.ZG compared to Adj.ZF (*p* = 0.022), Adj.ZR (*p* < 0.001) and aldosterone-producing lesion (APA *p* < 0.001). p21-immunoreactivity in APA cases was significantly higher in Adj.ZG than APA (*p* = 0.002), but not in Adj.ZF and Adj.ZR. In IHA, p16 was significantly higher in the ZG compared to ZR (*p* = 0.001) and aldosterone-producing lesion (*p* < 0.001) while p21 was not significantly different among them.

### 3.2. p16–p21 in CYP11B2-Positive (Aldosterone-Producing) and Negative Cells

**Figure 2:**
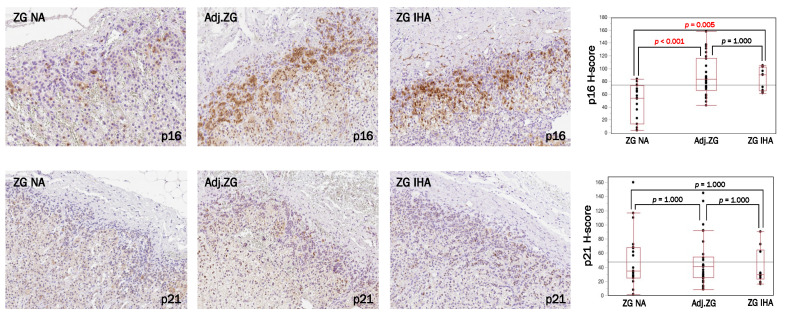
p16–p21 expression in CYP11B2-negative cells of ZG in NA, APA and IHA group. Immunohistochemical findings of p16- and p21-positive phenotypes in ZG of non-pathological adrenals (ZG NA), ZG adjacent to aldosterone-producing adrenocortical adenoma (Adj.ZG) and ZG of idiopathic hyperaldosteronism (ZG IHA). The figures demonstrated the increased p16-expression in PA patients’ zona glomerulosa (Adj.ZG and ZG IHA) compared to ZG NA, while no significant differences were detected using p21.

Results are summarized in Figure 2, Supplemental Figures S1 and S2. p16 immunoreactivity of adjacent ZGs of APA and IHA (Adj.ZG for APA cases and ZG IHA for IHA cases) were significantly more abundant than ZG of NA cases (*p* < 0.001; *p* = 0.005), but no such correlations were detected in p21 immunoreactivity.

### 3.3. γ. H2AX Immunolocalization in NA, APA and IHA

Results are summarized in Supplemental Figure S3. There were no significant differences of γH2AX immunoreactivity among normal adrenocortical layers (*p* = 0.193), as well as among CYP11B2-positive cells (*p* = 0.309). However, γH2AX immunoreactivity was significantly higher in the APA tumor area than in the adjacent zona glomerulosa (*p* < 0.01).

### 3.4. Intratumoral Heterogeneity of p16, p21 and CYP11B2 According to Cell Types in APAs

**Figure 3:**
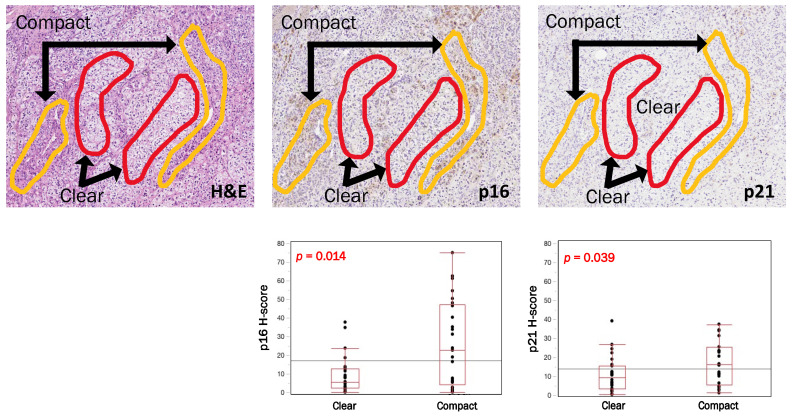
p16–p21 and CYP11B2 in clear and compact cells (intratumoral heterogeneity). Immunohistochemical findings of p16- and p21-positive phenotypes in clear and compact tumor cells of aldosterone-producing adrenocortical adenomas. H&E sections were used to carefully circumscribe the two different kinds of cells, and the IHC techniques highlighted a significantly higher expression of p16 and p21 among compact cells, compared to clear ones.

In APA cases we evaluated p16, p21 and CYP11B2 status in the total area of the tumor and particularly focused on intratumoral histological heterogeneity of tumor cells (clear and compact). Results are summarized in Figure 3. Compact tumor cells were more frequently positive for p16 and p21 compared to clear tumor cells (p16, *p* = 0.014; p21, *p* = 0.039), but there were no significant differences of CYP11B2 between these two cell types above (*p* = 0.085). We then explored the correlations between CYP11B2 and p16/p21 in clear or compact tumor cells separately, but no significant relationships were detected between those two parameters above (Supplemental Figure S4).

### 3.5. Comparative Analysis of p16 and p21 Immunoreactivity According to KCNJ5 Genotype (KCNJ5-Mutated vs. KCNJ5-Wild Type) in APAs

Results are summarized in Table 1. There were no significant differences between the status of *KCNJ5* and p16 but p21 was significantly more abundant in WT tumor (*p* = 0.008) and in clear cells (*p* = 0.029), compared to *KCNJ5*-mutated APAs. In addition, WT tumors tended to harbor higher CYP11B2 status (*p* = 0.060) and lower tumor size (*p* = 0.002). In addition, there were no significant differences of clinicopathological factors examined in this study between *KCNJ5*-mutated and WT APAs (Supplemental Table S5).

### 3.6. Correlations between Senescence Markers and Clinical Factors

**Figure 4:**
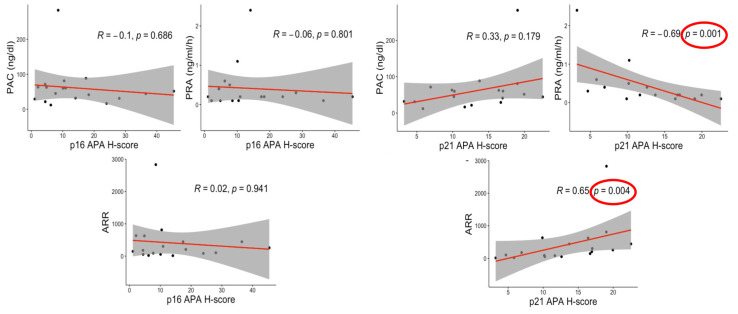
Correlation between Senescence markers and clinical factors in *KCNJ5* mutated group. The figure demonstrates the correlations between the main clinical factors in the APA group. Results highlighted significant correlations between p21 and PRA/ARR. No other significant correlations were detected in this study. PAC, plasma aldosterone concentration; PRA, plasma renin activity; ARR, aldosterone-to-renin ratio.

In APA, there were significant positive correlations between p21 (Figure 4) and the aldosterone-to-renin ratio (ARR) (R = 0.650; *p* = 0.004) and inverse ones between p21 and plasma renin activity (PRA) (R = −0.690; *p* = 0.001) in *KCNJ5*-mutated tumors. In addition, p21 status tended to be correlated with plasma aldosterone concentration (PAC) but this tendency did not reach statistical significance (R = 0.330; *p* = 0.179).

No significant correlations with p16 status were detected among the same subgroups and between p16 and p21 in WT APAs (Supplemental Figure S5. Results of IHA group are summarized in Supplemental Figure S6).

## 4. Discussion

To our knowledge, this is the first study focused on the status of cellular senescence in aldosterone-producing cells in both normal adrenal cortex and related disorders, including primary aldosteronism. The results provide novel insights into their association with adrenocortical zonation and the pathophysiology of aldosterone-producing disorders.

In normal human adrenal cortex, three zones were firstly morphologically described in 1866 [28]. Since then, the endocrinological importance of these layers has been largely established, but their formation or zonation, maintenance and regeneration throughout the life cycle have not been well explored [10,29]. Two different models/theories, the ‘centripetal’ [30,31] and the ‘zonal’ [32,33], have been proposed in previous literature. In the former model, adrenocortical cells were considered to originate from undifferentiated progenitor cells beneath the capsule and migrate inwardly, forming the ‘ZG to ZR’ gradient. In the latter model, each zone was self-maintained in an independent fashion from specific stem/progenitor cells. At this juncture, the centripetal theory has been considered rather more plausible, but definitive status has not been established yet [34]. Results of our present study firstly revealed that CYP11B2-negative or non-aldosterone-producing cells of the ZG were more senescent than other cortical cells, particularly in APA and IHA groups. These results could also be consistent with the ‘centripetal’ model, considering that the senescence gradient also followed the ZG to ZR direction. However, this correlation was not necessarily present in normal adrenal cortex. Therefore, further investigations are warranted to clarify the correlation between cell senescence and adrenal cortex development and zonation.

In addition, we firstly demonstrated that in the zona glomerulosa and adrenocortical neoplasms, CYP11B2-positive aldosterone-producing cells were less senescent than CYP11B2-negative or non-aldosterone producing cells. This finding could be probably explained by the increased cellular energy required for the aldosterone production. This process also involves complicated multiple cascades of reactions in both microsomes and mitochondria, and thus, avoidance of cellular senescence is considered mandatory to maintain the hormonal production and secretion in these cells above. In addition, among CYP11B2-negative cells of ZG, Adj.ZG and ZG IHA were more senescent than normal adrenals. This result could also reflect the lower aldosterone-producing ability of ZG cells under the suppressed PRA, which is consistent with results of the previous study demonstrating that Adj.ZG was characterized by suppression of HSD3B1/B2 and CYP11B2 expression compared to normal adrenals [35].

In addition, in order to further clarify the association between the status of DNA damage and the senescent status, we used γH2AX as a biomarker of DNA double-strand breaks caused by genotoxic agents such as UV or radiation [36]. DNA damage and replicative alteration could be a part of a senescent phenotype, although these phenotypes were reported to be not necessarily equivalent [37,38]. In this study, γH2AX immunolocalization was not concordant with those of the cell senescence biomarkers studied, including p16 and p21. However, of particular interest, γH2AX immunoreactivity was higher in the APA tumor area than in the adjacent zona glomerulosa, which could be related to the nature of neoplasms and consistent with the frequent genetic alteration detected in APA rather than in non-neoplastic adrenal glands [24,25,39], but further investigations are required for clarification, such as the exact cellular correlation between an induction of CYP11B2 and suppression of cell senescence.

We then explored the ‘intratumoral heterogeneity’ of cell senescence in APA by further studying CYP11B2 and p16-p21 status in clear and compact tumor cells circumscribed with care in the micrographic images. Results revealed significant differences between these two tumor cell types, i.e., clear cells were less senescent than compact tumor cells. In addition, clear cells in *KCNJ5*-mutated APAs were less senescent than those in WT APAs. Of particular interest, there were no significant differences of CYP11B2 status between these two different cell types of APA, suggesting that the status of cell senescence is by no means related to that of aldosterone production. However, these findings were consistent with previously reported results which demonstrated that clear cells were more active in aldosterone production in *KCNJ5*-mutated APAs [22] and harbored higher hormonal activity with lower senescence markers expression [18]. Regarding the correlation of cell senescence with genotypes, a significantly lower p21 was detected in *KCNJ5*-mutated tumors compared to WTs. The results above could be also explained by the fact that *KCNJ5*-mutated APAs had higher hormonal activity compared to WTs [26,40] and WT adenomas had more abundant compact cells structure [41,42].

When we evaluated the correlation between clinical findings and cell senescence, ARR and PRA were respectively positively and negatively correlated with p21 status in *KCNJ5*-mutated APA. Therefore, in situ aldosterone excess could also result in cellular senescence, possibly in a paracrine or autocrine fashion, with increased oxidative stress damages inflicted in the same manner as cortisol in CPAs [18,19], particularly in *KCNJ5*-mutated tumors. On the other hand, aldosterone excess was reported to damage heart, kidney, vessels and brain as direct aldosterone effects towards MR activation/oxidative stress, independently of blood pressure status [11,43,44,45]. In addition, this aldosterone-induced cellular senescence has already been reported in human and rat kidney proximal tubular cells with an oxidative stress/p53/p21-dependent pathway stimulated by mineralocorticoid receptor activation [46,47].

Intriguingly, another explanation could arise from the hypothesis of mitochondrial-senescence patterns and oxidative stress induced by aldosterone overproduction in tumor cells, but further investigations are required for clarification.

Limitations of this study included the descriptive nature and the absence of in vitro analysis as well as the unavailability of sufficient fresh frozen specimens to examine to further characterize cell senescence using SA-β Gal. The strengths of our study were the cohort selection, including surgical specimens from both APA and IHA patients and normal adrenals, and the novelty of this topic, which should provide new insights into the possible senolytic or senescence-induced therapies in aldosterone-related disorders.

## 5. Conclusions

In summary, CYP11B2-negative or non-aldosterone producing cells of the zona glomerulosa proved to be the most senescent group of among all the cortical cells, in terms of p16 and p21 status, especially among PA patients. The results also indicated that an induction of CYP11B2, the prerequisite of aldosterone production, could prevent cell senescence in order to maintain aldosterone production. In addition, CYP11B2-negative or non-aldosterone producing cells of ZG in non-pathological adrenal cortex in PA patients were more senescent than those of normal adrenal glands, possibly due to oxidative stress effects induced by autonomous aldosterone excess.

## Figures and Tables

**Table 1:** Cell senescence markers and intertumoral heterogeneity between *KCNJ5*-mutated and WT APAs.

Variable	KCNJ5 (*N* = 18)	WT (*N* = 12)	*p*-Value
p16 APA	10.27 (4.74; 19.72)	11.74 (5.62; 23.46)	0.688
p16 Clear Cells	6.52 (3.70; 14.15)	4.18 (2.62; 12.35)	0.467
p16 Compact Cells	31.16 (3.60; 47.44)	21.14 (5.94; 38.67)	0.718
p21 APA	13.18 (9.15; 17.51)	22.47 (13.25; 24.71)	0.008
p21 Clear Cells	6.60 (2.80; 12.17)	12.03 (7.00; 24.90)	0.029
p21 Compact Cells	10.44 (4.84; 30.98)	19.47 (12.25; 23.92)	0.305
CYP11B2 APA	15.90 (2.69; 35.12)	44.87 (13.42; 99.17)	0.060
CYP11B2 Clear Cells	15.45 (9.11; 33.48)	10.48 (3.39; 24.44)	0.764
CYP11B2 Compact Cells	44.54 (9.03; 52.43)	56.56 (25.98; 91.88)	0.349
Tumor Size (cm)	14.50 (9.75; 21.00)	7.50 (6.00; 10.00)	0.002

p16, p21 and CYP11B2 IHC all revealed that clear tumor cells of WT APAs abundantly expressed p21. In the related panel, WT APAs also displayed more abundant p21-expression and smaller size than *KCNJ5*-mutated ones. All the values were determined using H-Score as described in the Materials and Methods Section, using HALO^TM^ software. The values were reported with accuracy to one decimal point for all the cases examined. Clear and compact tumor cells were extrapolated within the tumor area as described in Materials and Methods Section. Red text means *p <* 0.05.

## Data Availability

The data that support the findings of this study are available from the corresponding author upon reasonable request.

## Supplementary Materials

The following are available online at https://www.mdpi.com/article/10.3390/biomedicines9050567/s1, Tables S1–S3. Clinicopathological characteristics of APA(S1), IHA(S2) and NA(S3) cohort; Table S4. IHC protocol and primary antibodies used for IHC; Table S5. Clinical factors comparisons in KCNJ5-mutated and WT APAs; Figure S1. p16–p21 status in CYP11B2-positive cells (aldosterone-producing). Figure S2. Correlations between CYP11B2 H-score and p16–p21 expression in NA, APA and IHA cohort. Figure S3. γH2AX immunolocalization in NA, APA and IHA. Figure S4. Correlations between CYP11B2 and senescence markers in clear and compact cells of APA group. Figure S5. Correlations between senescence markers and clinical factors in WT APAs. Figure S6. Correlations between senescence markers and clinical factors in IHA group.
