# Supplementary material for: Cellular Senescence in Human Aldosterone-Producing Adrenocortical Cells and Related Disorders
Source: Biomedicines. 2021 May 18;9(5):567. doi: 10.3390/biomedicines9050567 (PMC8158118; doi:10.3390/biomedicines9050567)
Supplement: Supplementary file 1 [file biomedicines-09-00567-s001.zip › biomedicines-1078964-supplementary.pdf]

biomedicines

Supplementary Material

Table S1. Clinicopathological characteristics of APA group.

Case	Gender/ Age	Res. Side	APA size (cm)	BMI (Kg/m²)	SBP (mmHg)	DBP (mmHg)	K (mmol/l)	PAC (ng/dl)	PRA (ng/mL/h)	ARR	eGFR (ml/min)	Genotype
1 APA	F/29	R	18	21,51	127	87	3,6	63,3	0,1	633	139	*KCNJ5*
2 APA	F/29	R	21	26,59	123	82	4,3	81,1	0,1	811	103	*KCNJ5*
3 APA	M/33	L	15	26,81	150	100	4,3	71,5	0,4	179	88	*KCNJ5*
4 APA	M/37	L	13	27,97	110	66	3,5	12,0	0,6	20	99	*KCNJ5*
5 APA	M/37	R	11	29,44	147	101	4,0	45,1	0,5	90	55	*KCNJ5*
6 APA	M/41	R	33	24,16	153	110	3,5	41,4	0,2	207	68	*KCNJ5*
7 APA	M/41	L	10	20,81	125	78	2,9	19,0	0,3	63	67	*WT*
8 APA	M/42	R	6	33,41	120	75	3,1	31,9	2,4	13	85	*KCNJ5*
9 APA	F/46	R	14	24,15	122	69	3,6	60,0	1,1	55	84	*KCNJ5*
10 APA	M/52	R	6	18,69	127	85	3,7	33,5	0,2	168	85	*WT*
11 APA	F/53	L	9	26,72	135	100	3,6	21,5	0,4	54	84	*KCNJ5*
12 APA	M/55	R	6	22,91	157	85	3,2	34,8	0,2	174	90	*WT*
13 APA	M/57	L	26	20,15	111	77	3,0	283,1	0,1	2831	51	*KCNJ5*
14 APA	M/59	R	17	26,73	140	93	3,7	88,4	0,2	442	68	*KCNJ5*
15 APA	F/60	L	10	28,86	146	83	3,8	33,2	0,1	332	76	*WT*
16 APA	M/60	R	5	24,56	131	66	4,3	43,8	0,2	219	83	*WT*
17 APA	M/61	L	10	20,62	183	115	3,4	31,1	0,3	104	71	*KCNJ5*
18 APA	M/61	L	9	23,8	169	104	4,0	27,0	0,4	68	88	*WT*
19 APA	M/62	L	8,5	21,51	164	102	4,3	16,6	0,2	83	29	*KCNJ5*
20 APA	M/63	L	7	26,52	132	85	4,0	19,8	0,1	198	63	*WT*
21 APA	M/64	L	5	23,59	118	80	4,1	29,2	0,2	146	76	*KCNJ5*
22 APA	M/64	L	10	26,56	162	99	4,0	41,9	0,2	210	93	*WT*
23 APA	M/67	L	12	23,03	163	104	3,9	51,7	0,2	259	67	*KCNJ5*
24 APA	M/67	L	6	20,49	118	83	3,4	28,2	0,3	94	76	*WT*
25 APA	F/68	R	24	22,76	122	81	4,2	62,6	0,1	626	29	*KCNJ5*
26 APA	F/68	L	18	27,47	152	84	3,1	44,4	0,1	444	75	*KCNJ5*
27 APA	M/70	R	21	25,34	132	86	3,3	60,4	0,2	302	41	*KCNJ5*
28 APA	M/71	R	8	25,23	136	71	2,9	59,5	0,2	298	44	*WT*
29 APA	F/72	L	13	23,78	150	86	3,8	58,0	0,2	290	88	*WT*
30 APA	M/73	R	7	27,01	137	73	3,3	41,6	0,2	208	57	*WT*

Table S2. Clinicopathological characteristics of IHA group.

Case	Gender / Age	Res. Side	SBP (mmHg)	DBP (mmHg)	K (mmol/l)	PAC (ng/dl)	PRA (ng/mL/h)	ARR
1 IHA	M/60	R	129	96	4,7	17,8	0,3	59,3
2 IHA	M/60	R	128	60	4,6	12,8	0,1	128
3 IHA	M/33	L	118	78	4,6	18,2	0,3	60,7
4 IHA	M/37	L	103	67	3,7	23,7	0,3	79
5 IHA	M/37	R	146	98	4,4	18,9	0,5	37,8

6 IHA	M/41	R	115	80	5	23,5	0,2	117,5
7 IHA	M/41	L	117	85	3,8	39,1	0,2	195,5
8 IHA	M/42	R	126	89	3,6	14,3	0,2	71,5
9 IHA	F/46	R	150	106	4,5	21,7	0,9	24,1
10 IHA	M/52	R	137	81	3,9	61,8	0,4	154,5

Table S3. Clinicopathological characteristics of NA group.

Case	Gender/Age	Res. Side	Diagnosis	Hypertension	eGFR (ml/min)
1 NA	F/33	R	Liver Sarcoma	NO	143.00
2 NA	M/41	R	Pancreatic Cancer	YES	88.00
3 NA	F/45	L	Pancreatic Cancer	NO	107.00
4 NA	M/51	L	Renal Cancer	NO	64.00
5 NA	M/54	R	Renal Cancer	NO	58.00
6 NA	F/54	R	Retroperitoneal tumor	NO	109.00
7 NA	M/58	L	Chronic Pancreatitis	NO	106.00
8 NA	M/61	R	Renal Cancer	YES	8.00
9 NA	F/61	R	Pancreatic Cancer	YES	84.00
10 NA	F/62	R	Renal Cancer	YES	4.00
11 NA	F/64	L	Renal Cancer	YES	61.00
12 NA	M/65	R	Renal Cancer	YES	56.00
13 NA	M/66	L	Renal Cancer	YES	65.00
14 NA	F/71	R	Pancreatic Cancer	NO	80.00
15 NA	M/73	L	Pancreatic Cancer	YES	91.00
16 NA	M/73	R	Renal Cancer	YES	48.00
17 NA	M76	L	Renal Cancer	YES	72.00
18 NA	F/77	L	Renal Cancer	YES	30.00
19 NA	M/78	L	Renal Cancer	YES	81.00

Clinicopathological characteristics of APA (S1), IHA (S2) and NA (S3) patients. Blood pressure levels for APA and IHA cohort were measured before adrenal vein sampling (AVS), after withdrawal of renin angiotensin aldosterone system (RAAS)-inhibitors, including mineralocorticoid receptor antagonists (MRAs). NA cohort definition of hypertension (YES/NO) was based on ESC/ESH 2018 Guidelines for the management of arterial hypertension. Res. Side, resected side; R, right and L, left; Age, age at surgery; BMI, body mass index; SBP, systolic blood pressure; DBP, diastolic blood pressure, K, potassium; PAC, plasma aldosterone concentration; PRA, plasma renin activity; ARR, aldosterone-to-renin ratio; eGFR, estimated glomerular filtration rate; WT, wild type.

Table S4. IHC protocols and primary antibodies.

Antibody	Antigen Retrieval Treatment	Isotype	Source/Reference	Control Tissue	Dilution
CYP11B2	Autoclave 121 °C 5min 50× Envision TM FLEX	Mouse IgG1 Monoclonal	Provided by Prof. Celso Gomez Sanchez	APA	1:500
p16	Autoclave 121 °C 5min, 0.05 M Citrate Buffer	Mouse IgG1 Monoclonal	BD Pharmingen	Colorectal Cancer	1:100
p21	Autoclave 121 °C 5min 0.05 M Citrate Buffer	Mouse IgG1 Monoclonal	BD Pharmingen	Colorectal Cancer	1:100
γH2AX	Autoclave 121°C 5min pH9 Buffer	Rabbit monoclonal	Cell Signaling Technology	Renal cell carcinoma	1:100

Antibodies and protocols used for IHC. All the antibodies were used at the same concentration as shown in the table for all other samples.

Table S5. Clinical factors comparisons in *KCNJ5*-mutated and WT APAs.

**Variable**	***KCNJ5* (*N* = 18)**	**WT (*N* = 12)**	***p*-value**
Gender (ref. Male)	12.00 (66.67)	10.00 (83.33)	0.419
Age at Surgery (y)	55.00 (37.00; 64.75)	62.00 (56.25; 70.00)	0.072
BMI (Kg/m²)	24.75 (22.45; 26.97)	24.18 (21.33; 26.55)	0.459
SBP (mmHg)	133.50 (121.5; 152.25)	136.50 (128.00; 155.25)	0.511
DBP (mmHg)	86.50 (79.25; 101.25)	84.00 (74.25; 85.75)	0.271
K+ (mmol/l)	3.60 (3.37; 4.12)	3.75 (3.22; 4.00)	0.671
PAC (ng/dl)	48.40 (30.62; 65.35)	34.15 (27.30; 43.32)	0.103
PRA (ng/mL/h)	0.20 (0.10; 0.42)	0.20 (0.20; 0.27)	0.640
ARR	192.9 (75.87; 489.50)	203.00 (112.37; 272.25)	0.849
eGFR (ml/min)	72.95 (53.86; 85.62)	79.83 (64.20; 88.11)	0.512

Clinical factors comparisons in *KCNJ5*-mutated and *KCNJ5*-wild type APAs. The value was reported with accuracy to one decimal point for all the cases analyzed. WT, wild type; BMI, body mass index; SBP, systolic blood pressure; DBP, diastolic blood pressure, K^+, potassium; PAC, plasma aldosterone concentration; PRA, plasma renin activity; ARR, aldosterone-to-renin ratio; eGFR, estimated glomerular filtration rate.

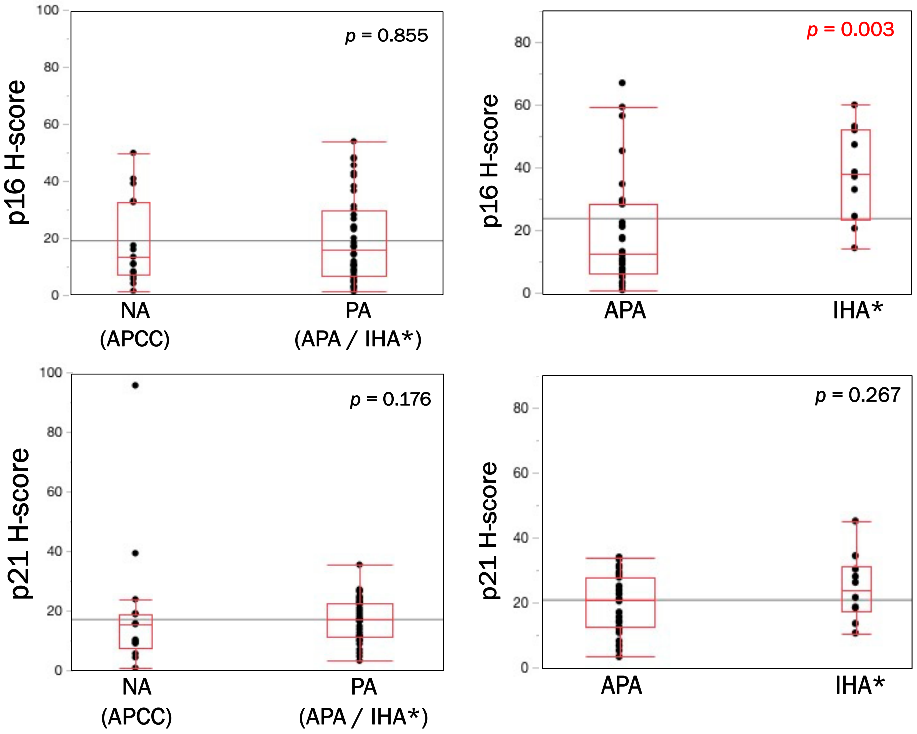

Figure S1. p16-p21 status in CYP11B2-positive (aldosterone-producing) cells. p16-p21 status in CYP11B2-positive cells. Firstly, we evaluated the p16-p21 status in aldosterone-producing or CYP11B2-positive cells between NA and PA cohorts considered together (APA and IHA). No significant differences were detected among those groups. We then analyzed p16 and p21 among PA cases and compared the results between APA and IHA. p16-immunoreactivity was significantly higher in IHA than APA, but not p21. (*) is used to identify the CYP11B2-positive lesion responsible for aldosterone overproduction in the IHA group.

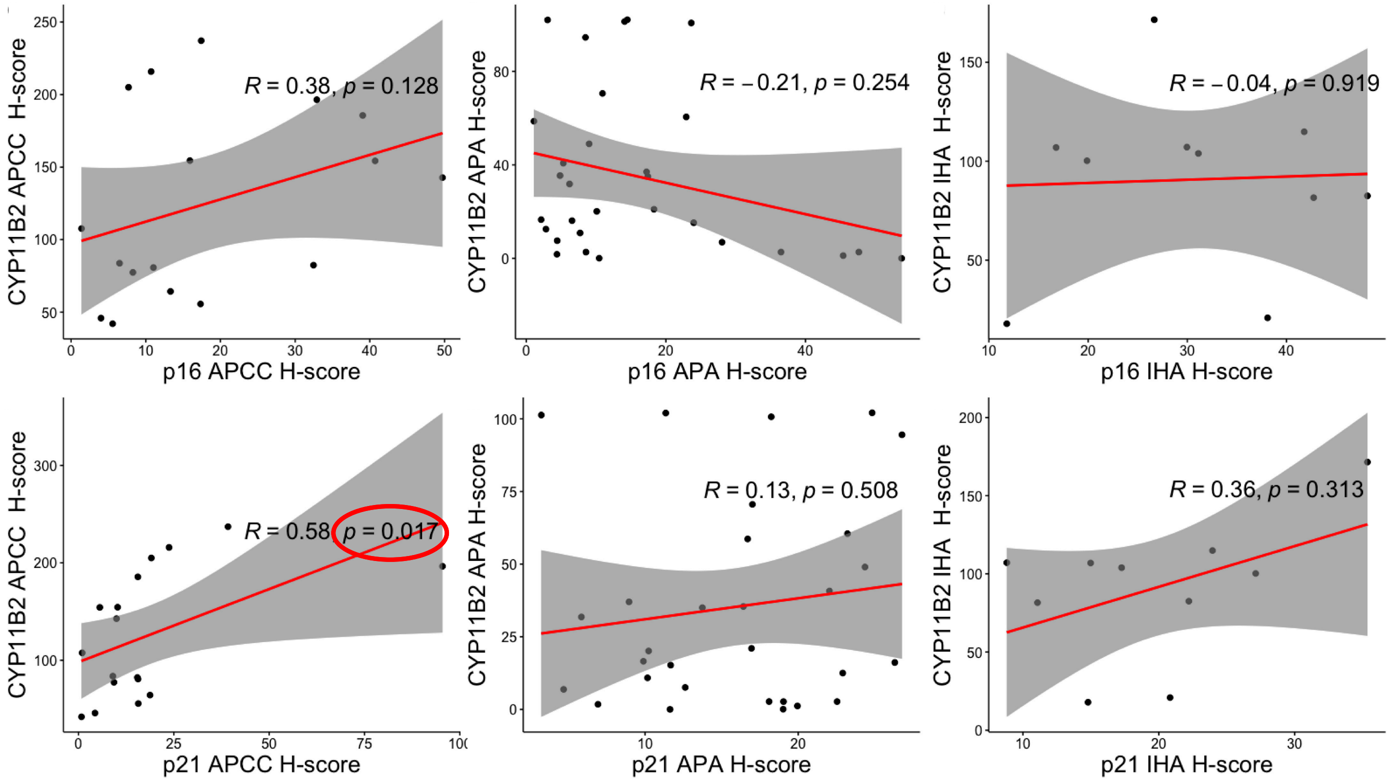

Figure S2. Correlations between CYP11B2 H-score and p16-p21 expression in NA, APA and IHA cohort. Correlations between CYP11B2 H-score and p16-p21 expression in NA, APA and IHA cohort. The figure shows the correlations between the CYP11B2 H-score and senescence markers in NA, APA and IHA group. The results show a significant correlation between CYP11B2 and p21 expression in APCC. The other results did not highlight significant relationships.

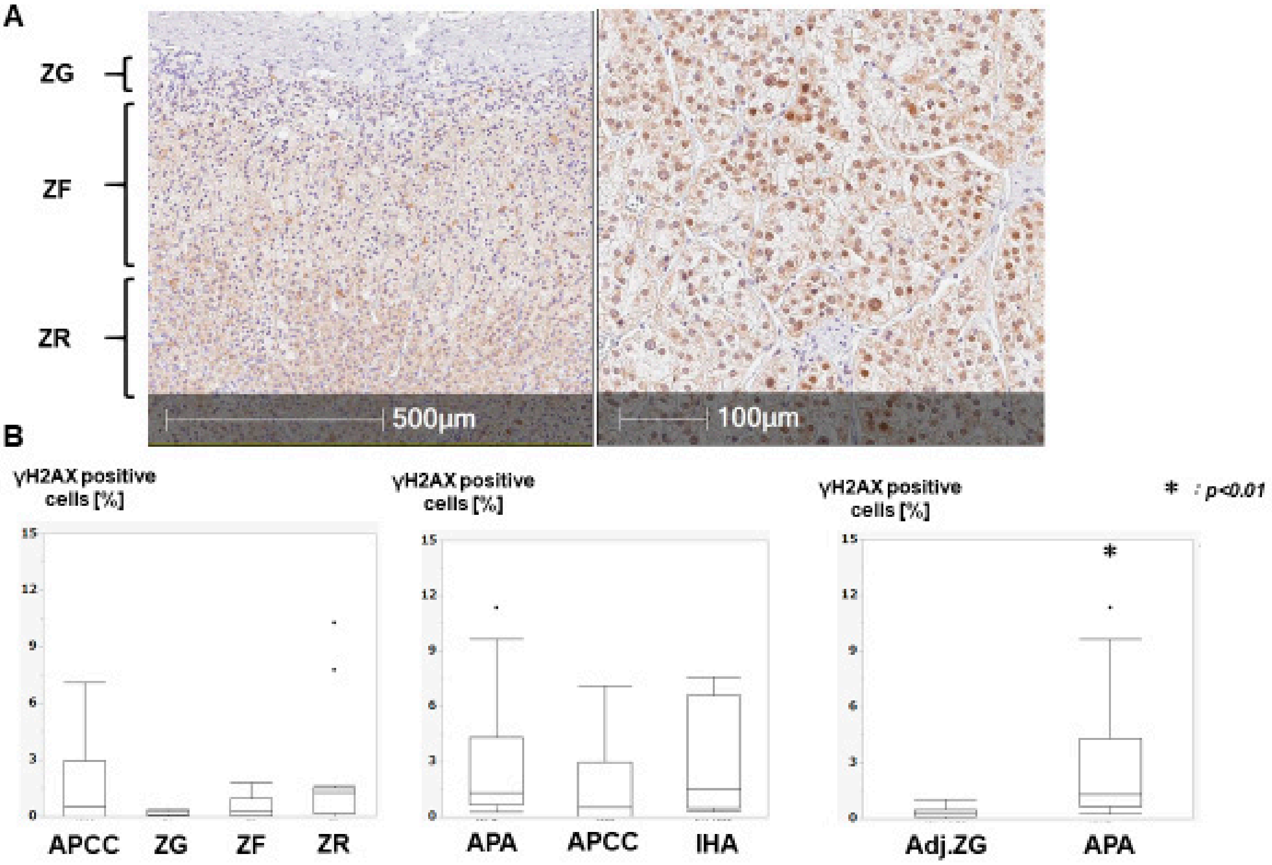

Figure S3. γH2AX immunolocalization in NA, APA and IHA. APA: aldosterone-producing adenoma (tumor), APCC: aldosterone-producing (CYP11B2-positive) cell cluster, ZG: zona glomerulosa (CYP11B2-negative), ZF: zona fasciculata, ZR: zona reticularis, IHA: idiopathic aldosteronism (CYP11B2-positive cell clusters), Adj. ZG: adjacent zona glomerulosa to APA. "*" means $p < 0.01$. (**A**): Representative images of γH2AX immunohistochemistry in NA (left) and APA (right). (**B**): Comparative analysis of γH2AX immunoreactivity in NA (left), aldosterone-producing (CYP11B2-positive) cells (middle) and APA (right). No significant difference of γH2AX immunoreactivity was detected in NA and in CYP11B1-positive cells. However, γH2AX immunoreactivity was significantly higher in the tumor area of APA than in the adjacent ZG.

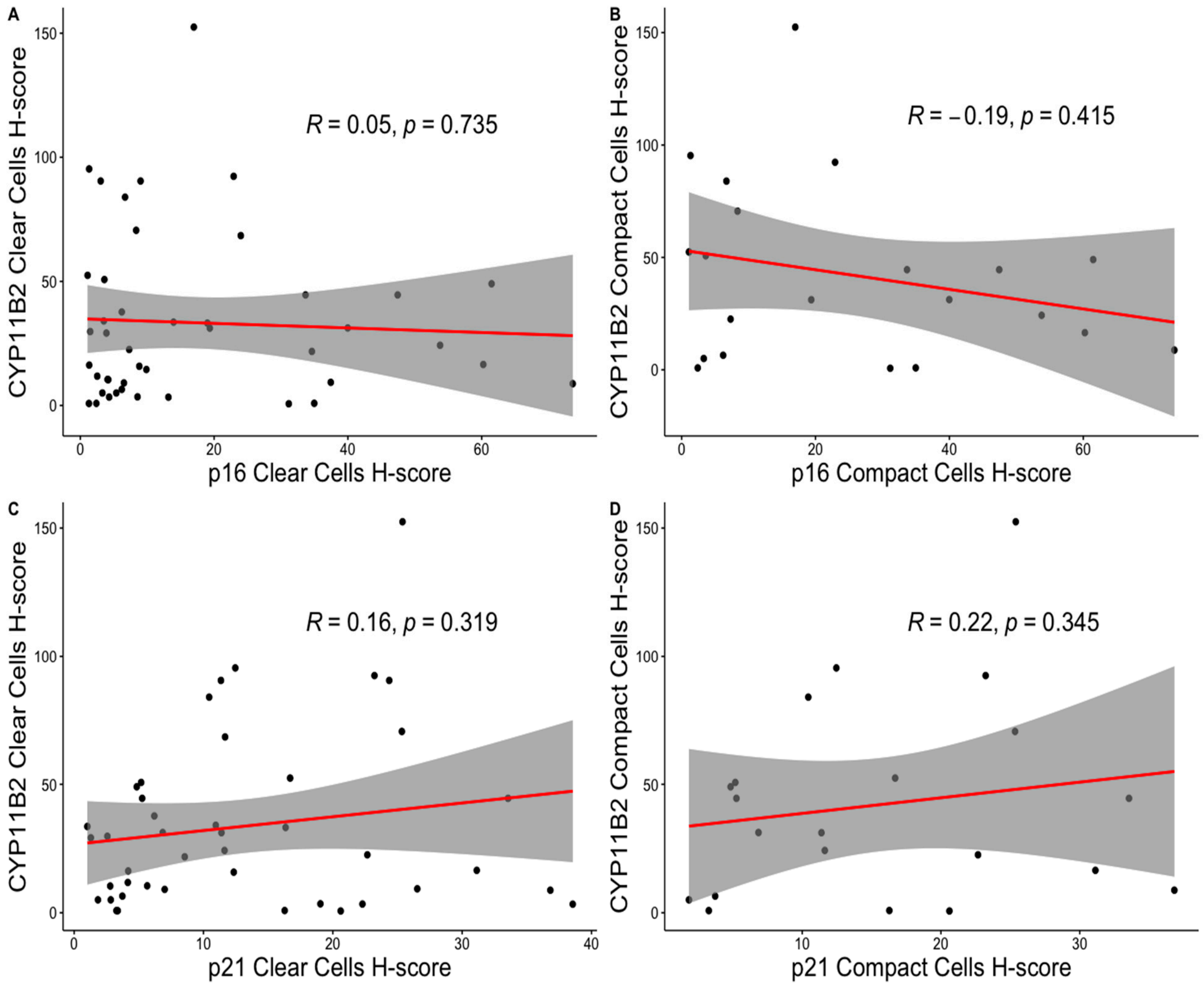

Figure S4. Correlations between CYP11B2 and senescence markers in clear and compact cells in APA group. The figure shows the correlations between CYP11B2 and p16-p21 expression in clear and compact cells of the APA group. The results did not show significant correlations. All the p16 and p21 values were determined using H-Score as described in the Materials and Methods Section, using HALO™ software.

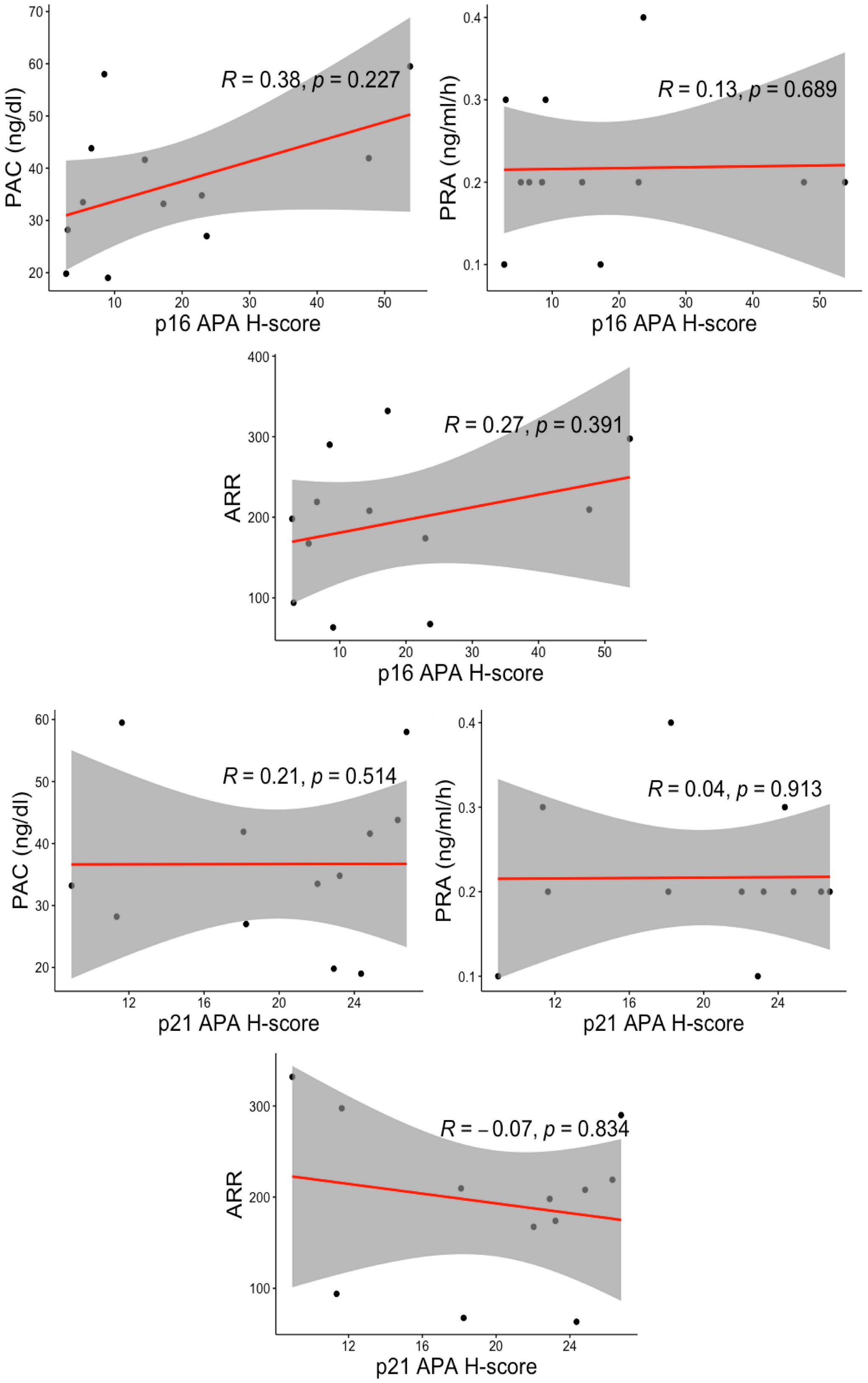

Figure S5. Correlations between senescence markers and clinical factors in WT APAs. Correlation between Senescence markers and clinical factors in KCNJ5-wild type group. The figure shows the correlations with the main clinical factors in the APA group. The results did not show significant correlations. PAC, plasma aldosterone concentration; PRA, plasma renin activity; ARR, aldosterone-to-renin ratio. All the p21 values were determined using H-Score as described in the Materials and Methods Section, using HALO™ software.

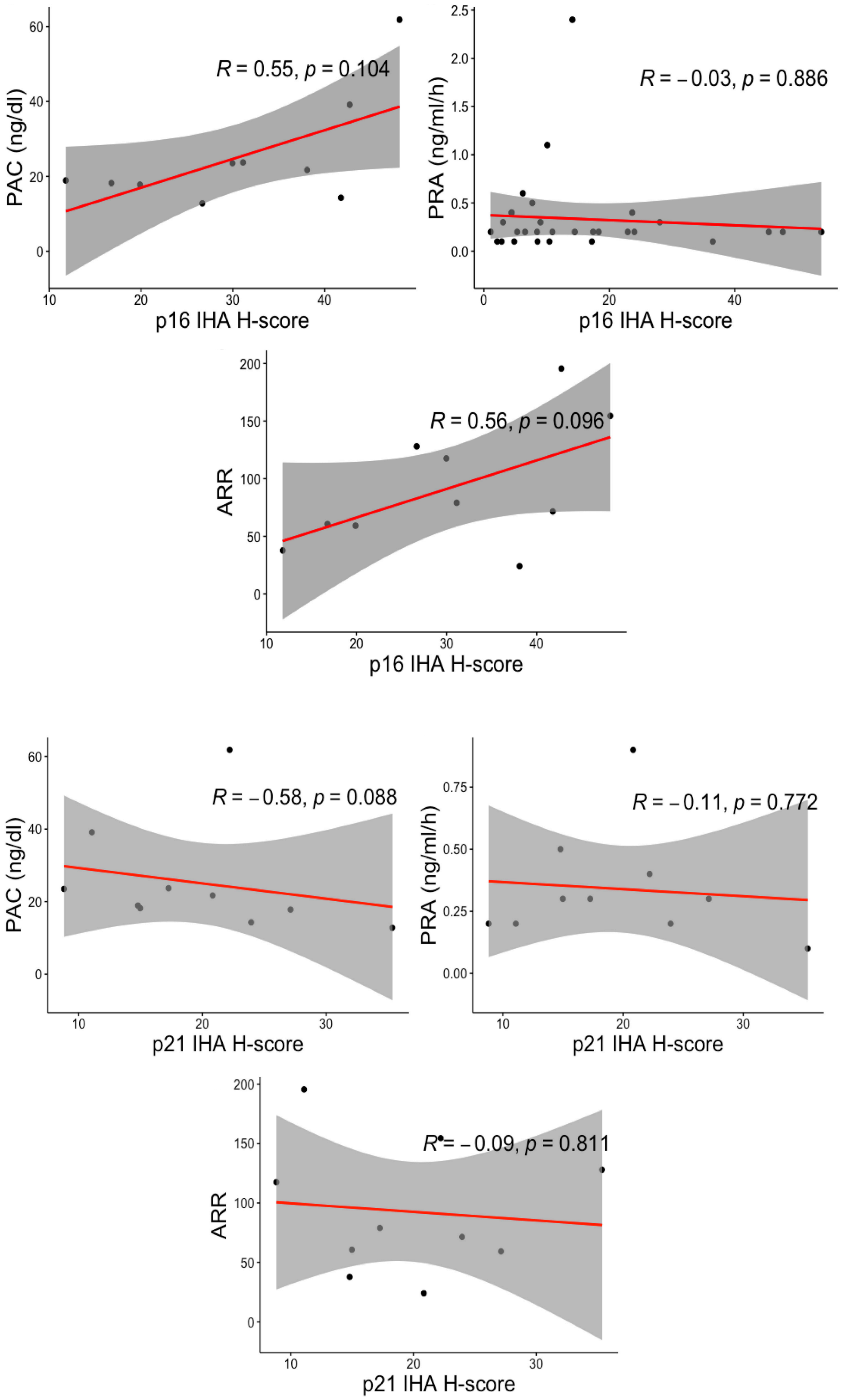

Figure S6. Correlations between senescence markers and clinical factors in IHA group. The figure shows the correlations with the main clinical factors in IHA patients. The results did not show significant correlations. PAC, plasma aldosterone concentration; PRA, plasma renin activity; ARR, aldosterone-to-renin ratio. All the p21 values were determined using H-Score as described in the Materials and Methods Section, using HALO™ software.
